# COVID-19 in a Patient With Severe Eosinophilic Asthma on Benralizumab Therapy: A Case Report and Review of Literature

**DOI:** 10.7759/cureus.20644

**Published:** 2021-12-23

**Authors:** Osamu Matsuno, Seijiro Minamoto

**Affiliations:** 1 Department of Medicine for Allergic Disease, Osaka Habikino Medical Center, Habikino, JPN

**Keywords:** il-5, eosinophil, severe asthma, covid-19, benralizumab

## Abstract

Patients with coronavirus disease 2019 (COVID-19) can develop eosinopenia. Eosinophils have various functions, including immunoregulation and antiviral activity, in addition to modulation of an inflammatory reaction. Benralizumab is an anti-interleukin-5Rα monoclonal antibody that selectively depletes eosinophils through enhanced antibody-dependent cell-mediated cytotoxicity. Whether eosinophil depletion affects COVID-19 prognosis is yet to be elucidated. Here, we present a case of a 60-year-old patient with severe asthma on benralizumab therapy, who tested positive for an acute respiratory syndrome coronavirus 2 (SARS-CoV-2) infection. The patient experienced an asymptomatic COVID-19 course without deterioration of asthma control. Eosinophil depletion did not contribute to a deterioration of the clinical status. Comorbidities play a major role in the severity of COVID-19 in patients with asthma. The findings of our case and a literature review revealed that benralizumab therapy is not associated with a significant negative impact on the disease course in COVID-19 patients.

## Introduction

Asthma has not been identified as a risk factor for more severe outcomes in the coronavirus disease (COVID-19) in any of the larger case series reported to date [1]. Benralizumab has increasingly garnered interest in the treatment of severe eosinophilic asthma in the setting of COVID-19. Benralizumab is a monoclonal antibody that is an IL-5-receptor-alpha blocker that depletes eosinophils through antibody-dependent cell-mediated cytotoxicity [2]. However, eosinopenia has been observed in COVID-19 patients and may be a risk factor for worse disease outcomes [3,4], although the mechanisms underlying this phenomenon are not fully understood. Benralizumab may be a useful tool to clarify the protective or exacerbating role of eosinophils in COVID-19, but there are few reports of the use of benralizumab to treat severe asthma in COVID-19 [5-9] and none from non-Western countries. It is unknown whether benralizumab impacts disease activity, including asthma control, in COVID-19. We report a case of severe asthma in a benralizumab-treated Japanese patient with asymptomatic severe acute respiratory syndrome coronavirus 2 (SARS-CoV-2) infection. Moreover, we reviewed the English literature on COVID-19 in benralizumab-treated patients with severe eosinophilic asthma.

## Case presentation

We present the case of a 60-year-old woman (body mass index 22.2 kg/m2) characterized by frequent asthma exacerbations, asthma control test (ACT) of 19; severe airflow limitation with expiratory volume in 1 second (%FEV1) of 38.5%; productive cough (sputum ++); eosinophilia (eosinophil count 1,510/μL); and a fraction of exhaled nitric oxide (FeNO) of 57 ppb. Benralizumab (30 mg, subcutaneously, every four weeks for the initial three doses, and every eight weeks thereafter) was initiated in March 2019, which led to a rapid and lasting improvement in the asthma control (ACT 4; 39.4% improvement in %FEV1) as well as complete depletion of blood eosinophils after 4 weeks. FeNO, however, was elevated to 77 ppb. The patient was treated with budesonide/formoterol eight puffs (1,280 μg/36 μg)/day, tiotropium two puffs/(5 μg) day, montelukast 10 mg/day, and short bursts of systemic corticosteroids. On May 4, 2021, the patient’s husband developed fever, and the patient tested positive for SARS-CoV-2 on reverse transcriptase-polymerase chain reaction testing of a nasopharyngeal swab, despite asymptomatic of COVID-19. The patient’s asthma was well-controlled (ACT 25), but her blood eosinophil count and pulmonary function (spirometry) were not examined. Three weeks after the detection of the asymptomatic SARS-CoV-2 infection, the patient’s eosinophil count was 0/μL, FeNO was 62 ppb, and %FEV1 was 64.5%, without wheezing on chest auscultation, and with good asthma control (ACT 25). IgG antibodies against the SARS-CoV-2 spike protein were positive (261.1 AU/mL) without a history of consistent manifestations, indicating past asymptomatic infection (Table 1).

**Table 1:** Demographics and clinical characteristics of patients with severe asthma and COVID-19 on benralizumab treatment AZM, azithromycin; CRSwNP, chronic rhinosinusitis with nasal polyps; EOM, eosinophilic  otitis media; HT, hypertension; NA, not available; OCS, oral corticosteroids; LVFX, Levofloxacin; SAS, sleep apnea syndrome

Pt	Sex	Age	Benralizumab duration (Mo)	COVID-19 confirmation	Comorbidities & known risk factors for severe COVID-19	Symptoms	Asthma exacerbation	Pneumonia	Treatment	Patient outcome	Reference
1	M	41	19	PCR	CRSwNP	Fever, Dyspnea, Back pain	(-)	(-)	Ibuprofen	Alive	Renner A et al. [5]
2	F	64	NA	PCR	Obesity	Fever, Dyspnea,	(+)	(+)	AZM, OCS	ICU, Alive	Kroes JA et al. [6]
						Migraine, Myalgia,					
3	F	56	NA	PCR	Bronchiectasis	Fever, Dyspnea, Arthralgia, Myalgia,	(-)	(-)	LVFX,OCS	Alive	García-Moguel et al. [7]
											4	M	62	21	Clinical	CRSwNP, bronchiectasis, SAS, and obesity	Fever, Cough,	(-)	(+)	LVFX, AZM, Hydroxychloroquine, and Amoxcillin	Alive	
5	M	66	16	PCR	CRSwNP	Fever, Anosmia, Fatigue	(-)	(-)	(-)	Alive	Renner et al. [8]
6	F	65-70	NA	PCR	Obesity	NA	(-)	NA	OCS	ICU, Alive	Eger et al. [9]
7	M	45-50	NA	PCR	NA	NA	(-)	NA	OCS	Alive	
8	F	60	38	PCR	HT, EOM	Asymptomatic	(-)	(-)	(-)	Alive	Our case

## Discussion

Viral infections of the upper and lower airways have been associated with the development and exacerbation of allergic disease [10]. Respiratory viruses including rhinovirus, respiratory syncytial virus, and influenza virus are common triggers of viral-induced asthma exacerbations, whereas coronaviruses are far less common triggers for acute asthma exacerbations [11]. 

Zhu et al. reported that the risk of severe COVID-19 was not significantly elevated in patients with allergic asthma. In contrast, adults with asthma had a higher risk of severe COVID-19, which was driven by the increased risk in patients with nonallergic asthma [12]. Airway and blood eosinophilia are biomarkers for allergic asthma and severe nonatopic late-onset asthma. Eosinophils have both potent proinflammatory effects in asthma and antiviral properties [13].

Eosinophils are considered contributing to an antiviral role by the release of specific cytotoxic granular constituents (ECP, EDN, and EPO), superoxide, and nitric oxide generation [14]. It is unknown whether eosinophils depletion induced by benralizumab impacts disease activity, including asthma control, in COVID-19.

Recent guidelines recommend continuing biologics (as clinically indicated) to maintain asthma control during SARS-CoV-2 infection [15]. Information on the course of COVID-19 in benralizumab-treated patients with severe asthma is scarce. Our review of the literature yielded five reports, comprising descriptions of SARS-CoV-2 infection in a total of seven patients from Western countries who were receiving benralizumab therapy for severe asthma. Our case is the first one to be reported from an Eastern country and presents an asymptomatic patient. In all these patients, including our patient, the most commonly reported comorbidities were chronic rhinosinusitis with nasal polyps (3/8) and obesity (3/8); asthma exacerbation was observed in only one patient (1/8), whereas pneumonia was observed in two patients (2/8); and oral corticosteroids (OCSs) were most frequently used for COVID-19-related symptoms (4/8). None of the patients died. It is unknown whether SARS-CoV-2 infection triggers acute asthma exacerbations in severe asthma patients receiving benralizumab therapy. Although these eight patients were receiving benralizumab, two patients who had recognizable risk factors (i.e., old age, obesity) for severe COVID-19 outcomes required intubation and mechanical ventilation in the intensive care unit and eventually recovered [16,17].

Age is one of the greatest risk factors for hospitalization due to COVID-19 in patients with asthma [16]. Obesity is a risk factor for severe outcomes in critically ill COVID-19 patients. Obese patients [A3] have a longer hospital stay and a higher mortality risk [17]. The known risk factors for severe COVID-19, such as age and obesity, are relatively common in patients with severe asthma due to high OCS exposure, and it may be difficult to distinguish which of the following risk factors contributes most to severe COVID-19: the severity of asthma, the use of biologics, or OCS-induced comorbidities. Our patient experienced an asymptomatic disease course without deterioration of asthma control. Eosinophil depletion did not contribute to a deterioration of the clinical status. The literature review as well as our case shows that benralizumab therapy is not associated with a significant negative impact on the disease course in COVID-19 patients.

## Conclusions

In view of the abovementioned facts, obesity and age may contribute to the severity of COVID-19 in patients with asthma who are treated with benralizumab, rather than the effect of eosinopenia. Patients with asthma and COVID-19 were at increased risk due to any other possible risk factors. Benralizumab is generally safe. Patients with asthma without any other possible risk factors may not be considered high risk for severe COVID-19 outcomes.
